# Supplementary material for: Causal Relationship between Aging and Anorexia Nervosa: A White-Matter-Microstructure-Mediated Mendelian Randomization Analysis
Source: Biomedicines. 2024 Aug 16;12(8):1874. doi: 10.3390/biomedicines12081874 (PMC11351342; doi:10.3390/biomedicines12081874)
Supplement: Supplementary file 1 [file biomedicines-12-01874-s001.zip › Supplementary Figure/Figure S8/Leaveoneout plot of MR analysis for TL to ubm-b-1502.pdf]

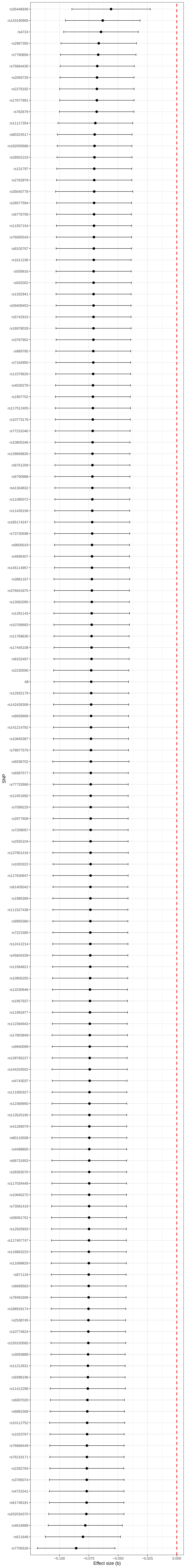
rs35446936
rs143190905
rs4724
rs2967355
rs7790856
rs75664430
rs2056726
rs2276182
rs17677991
rs762679
rs11117354
rs80324517
rs182059586
rs28502153
rs131797
rs2763979
rs35640778
rs28577594
rs6776756
rs11557154
rs76065543
rs8105767
rs1611236
rs939916
rs932002
rs1332941
rs59409453
rs5742915
rs16978028
rs3767952
rs869785
rs7164950
rs11579626
rs4530278
rs1907702
rs117512405
rs10773176
rs77231040
rs10805346
rs139669835
rs6751209
rs6790988
rs41304832
rs11085072
rs11426156
rs185174247
rs73730598
rs9600019
rs4695407
rs145114957
rs3891167
rs376641875
rs13062095
rs1291143
rs10768683
rs11769630
rs17445108
rs8102497
rs2230590
All
rs12932179
rs142426306
rs6659669
rs141214782
rs10845387
rs79977579
rs6536702
rs6587577
rs77732866
rs12451892
rs7099229
rs2977608
rs7209057
rs2555104
rs137901416
rs1003322
rs117630647
rs61405042
rs1985369
rs111527438
rs9955360
rs7221585
rs12412214
rs45604339
rs11584821
rs10905255
rs13230646
rs1957937
rs11991877
rs112394943
rs17803849
rs9940099
rs139795227
rs144204502
rs4743037
rs111950327
rs12369950
rs113525195
rs41269079
rs80116508
rs4498805
rs66731853
rs28363070
rs117034449
rs10840270
rs73581419
rs56061761
rs12925933
rs117407747
rs116863223
rs11699829
rs871134
rs6669563
rs78491606
rs188918174
rs2538745
rs10774624
rs150150565
rs3093888
rs11212631
rs9398196
rs11412296
rs6007020
rs6881568
rs10112752
rs1023767
rs76666449
rs76219171
rs2282764
rs3785074
rs4731541
rs61748181
rs202034370
rs4616688
rs611646
rs7705526
SNP
−0.100
−0.075
−0.050
−0.025
0.000
Effect size (b)
